# PP43 Factors Associated With Interrupting Treatment With Somatropin: Analysis Of Real-World Data From Argentina

**DOI:** 10.1017/S0266462325102626

**Published:** 2025-12-29

**Authors:** Jorge Elgart, Carolina La Mura, Cintia Campelo, Lucila Rey-Ares

## Abstract

**Introduction:**

Lack of persistence and adherence to growth hormone therapy negatively impacts growth outcomes. Real-world international studies report that a significant proportion of children discontinue somatropin treatment. However, this information is not available for Argentina. The aim of this study was to describe the persistence and reasons for discontinuation of somatropin treatment in pediatric patients in the real-world setting of Argentina.

**Methods:**

This retrospective cohort study analyzed data from the Anhelos program (Argentina) to evaluate somatropin treatment in children under 16 years of age. Persistence and treatment discontinuation were measured, with causes classified as medical or non-medical. Statistical methods were used to analyze predictive factors such as sex, age, diagnosis, region, and healthcare coverage. Persistence was analyzed using Kaplan-Meier, while discontinuation was studied through logistic regression and Cox proportional hazards models. SPSS v26.0 was used, with significance defined as p-value≤0.05.

**Results:**

We analyzed 1,159 patients, 42.8 percent of whom were female; 58.7 percent were aged eight years or younger. A total of 56.7 percent discontinued somatropin treatment at least once. No significant differences were observed in sex, age, or region between those who interrupted treatment and those who did not. However, differences were found based on diagnosis and healthcare coverage. Non-medical causes accounted for 64.6 percent of treatment discontinuations, primarily due to delivery issues. Public healthcare coverage was associated with a higher likelihood of discontinuation and lower treatment persistence than social security or private health insurance coverage.

**Conclusions:**

Our data suggested a high rate of at least one treatment discontinuation among pediatric patients in Argentina receiving somatropin, primarily driven by non-medical causes.

